# Premier cas de mpox dépisté au Mali dans une zone d’orpaillage en 2025 grâce à la télédermatologie

**DOI:** 10.48327/mtsi.v6i3.2026.786

**Published:** 2026-09-11

**Authors:** Dramane TRAORÉ, Bekaye TRAORÉ, Allaye Ebe ARAMA, Moussa SOUGANÉ, Mahmoud CISSÉ, Hamidou Souleymane TAPILY, dou SaïTOURÉ, Daouda MOLBA, Mouhamoudou Habib TRAORÉ, Mahamadou Abdou DIALLO, Mirana Michelle RANDRIAMBELONORO, Aziza MERZOUKI, hima IbréGUINDO, Cheick Oumar BAGAYOKO, Ousmane FAYE

**Affiliations:** 1 Centre d’expertise et de recherche en télémédecine et e-santé (CERTES), Bamako, Mali; 2 Hôpital de dermatologie de Bamako (HDB), Rue 315, Bamako, Mali; 3 Centre de santé communautaire (CSCom) de Kourémalé, Kourémalé, Mali; 4 Centre de santé de référence (CSRéf) de Kangaba, Mali; 5 Université des sciences, des techniques et des technologies de Bamako (USTTB), Mali; 6 Hôpital régional de Sikasso, Mali; 7 Institut national de santé publique (INSP), Avenue Kasse Keita, Bamako, Mali; 8 Geneva Digital Health Hub (gdhub), G6-03 Chemin des Mines 91202 Genève, Suisse

**Keywords:** Mpox, Télédermatologie, Maladie émergente, Surveillance épidémiologique, Orpaillage, Kourémalé, Mali, Afrique subsaharienne, Mpox, Teledermatology, Emerging disease, Epidemiological surveillance, Alluvial gold mining, Kourémalé, Mali, Sub-Saharan Africa

## Abstract

**Introduction:**

Le mpox est une zoonose virale émergente causée par le virus mpox, appartenant au genre *Orthopoxvirus*. Son diagno stic repose en grande partie sur la reconnaissance de manifestations cutanées caractéristiques, conférant à la dermatologie un rôle central dans sa détection précoce. Nous rapportons le premier cas confirmé de mpox au Mali, identifié grâce à une téléconsultation dermatologique dans une zone frontalière à forte mobilité humaine.

**Méthode:**

Cette observation a été réalisée dans le cadre d’un programme national de téléconsultation coordonné par le Centre d’expertise et de recherche en télémédecine et e-santé (CERTES). Les données cliniques et les photographies des lésions ont été transmises via la plateforme sécurisée HUG@home pour téléexpertise dermatologique. La confirmation diagnostique a reposé sur une PCR en temps réel réalisée sur un prélèvement cutané au laboratoire national de référence, complétée par un séquençage viral.

**Résultats:**

Un homme de 35 ans travaillant dans l’orpaillage artisanal dans une zone frontalière entre le Mali et la Guinée a consulté pour un syndrome fébrile associé à une éruption cutanée évoluant depuis trois semaines. L’examen dermatologique à distance a mis en évidence des lésions papulo-vésiculo-pustuleuses ombiliquées évocatrices de mpox. Cette suspicion a conduit à l’isolement immédiat du patient et à la réalisation d’investigations biologiques. Le diagnostic a été confirmé par PCR 72 heures après la téléconsultation et le séquençage a identifié un virus appartenant au clade IIb. Une enquête épidémiologique a permis d’identifier et de suivre 48 contacts pendant 21 jours sans apparition de cas secondaire durant cette période. Toutefois, trois autres cas confirmés ont été détectés ultérieurement dans la même localité, dont un agent de santé exposé lors de la prise en charge du cas index. L’évolution clinique du patient a été favorable avec une guérison complète après quatre semaines de suivi.

**Conclusion:**

Ce premier cas de mpox confirmé au Mali, détecté grâce à la télédermatologie, a conduit à l’identification ultérieure de cas supplémentaires dans la même localité, suggérant une circulation locale du virus. Cette observation souligne le potentiel de la téléexpertise dermatologique pour la détection précoce des maladies émergentes à expression cutanée et le renforcement de la surveillance épidémiologique dans les contextes à ressources limitées. Des études complémentaires sont nécessaires pour évaluer son impact sur les performances diagnostiques, la prise en charge des patients et les systèmes de surveillance à plus grande échelle.

## Abstract (en)

**Introduction:**

Mpox, an emerging viral zoonosis, is caused by the mpox virus, which belongs to the *Orthopoxvirus* genus. Diagnosis largely relies on recognizing characteristic skin manifestations, which gives dermatology a central role in early detection. Here, we report the first confirmed case of mpox in Mali, which was identified through a dermatological teleconsultation in an area with high human mobility along the border.

**Methods:**

This case was identified as part of a national teleconsultation program coordinated by the Center for Expertise and Research in Telemedicine and e-Health (CERTES). Clinical data and photographs of the lesions were transmitted via the secure HUG@home platform for dermatological tele-expertise. Diagnostic confirmation was based on real-time PCR performed on a skin specimen at the national reference laboratory, supplemented by viral sequencing.

**Results:**

A 35-year-old man working in artisanal gold mining in a border area between Mali and Guinea presented with a three-week-long rash associated with a febrile syndrome. A remote dermatological examination revealed umbilicated papulovesicular-pustular lesions, which are suggestive of mpox. This led to the patient’s immediate isolation and initiation of laboratory testing. The diagnosis was confirmed by PCR 72 hours after the teleconsultation, and sequencing identified a clade IIb virus. An epidemiological investigation identified and monitored 48 contacts for 21 days; no secondary cases emerged during that period. However, three other confirmed cases were subsequently detected in the same area, including a healthcare worker who was exposed while caring for the initial case. The patient made a full recovery after four weeks of follow-up.

**Conclusion:**

The first confirmed case of monkeypox in Mali was detected through teledermatology and led to the identification of additional cases in the same area, suggesting the virus is circulating locally. This observation underscores the potential of dermatological tele-expertise in the early detection of emerging cutaneous diseases and in strengthening epidemiological surveillance in settings with limited resources. Further studies are needed to evaluate the impact of dermatological tele-expertise on diagnostic performance, patient care, and surveillance systems on a larger scale.

**Figure:**
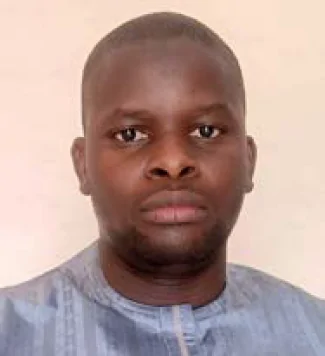

## Introduction

Le mpox anciennement appelé Monkeypox ou variole du singe est une maladie virale zoonotique causée par un *Orthopoxvirus*, présentant des similitudes cliniques avec la variole humaine mais généralement moins sévère [8]. Sur le plan clinique, l’infection se traduit essentiellement par des lésions cutanéo-muqueuses. La transmission à l’être humain peut survenir à la suite d’un contact direct avec un animal infecté ou avec une personne infectée [7]. Le premier cas humain a été décrit en 1970 par Ladnij *et al.* chez un garçon de neuf mois non vacciné contre la variole à Basankusu, une province équatoriale de la République démocratique du Congo (RDC) pendant les efforts d’éradication de la variole [10]. Une première épidémie a été rapportée en RDC en 1997 après recensement de 88 cas d’éruptions pustuleuses fébriles, parmi lesquels le virus a pu être isolé chez 7 patients [9].

Le mpox a longtemps été endémique dans les zones forestières de l’Afrique de l’Ouest et centrale [4]. Depuis 2022 cette maladie s’est rapidement propagée à travers le monde avec environ 81 225 cas confirmés dans plus de 100 pays[1]. Bien qu’aucun cas de mpox n’ait été officiellement notifié au Mali à ce jour, des cas ont été rapportés dans plusieurs pays voisins, notamment en Côte d’Ivoire et en Guinée Conakry [17], soulignant le risque potentiel d’introduction du virus sur le territoire national. Dans ce contexte de circulation régionale du virus, la détection précoce des cas suspects et l’accès à un diagnostic de confirmation, en particulier dans les zones périphériques à ressources limitées, demeurent des défis majeurs pour le système de santé. La télédermatologie existe au Mali depuis 2015 et contribue à l’amélioration de la prise en charge des affections cutanées dans les centres de santé périphériques [6]. En facilitant l’accès à une expertise spécialisée à distance, elle réduit les inégalités d’accès aux soins dermatologiques entre les zones urbaines et rurales. L’utilisation des technologies numériques au service de la santé publique peut constituer un outil de prise en charge médicale mais aussi un véritable levier stratégique pour renforcer la surveillance épidémiologique de certaines maladies dans les zones éloignées et difficiles d’accès [2,3]. Nous rapportons ici le premier cas de mpox du Mali dépisté *via* la téléconsultation.

## Méthodes

La téléconsultation dermatologique est assurée en mode synchrone à travers la plateforme sécurisée HUG@home. Celleci permet une communication audiovisuelle en temps réel entre le patient, pris en charge dans une structure sanitaire périphérique par un agent de santé, et un dermatologue expert, facilitant ainsi l’évaluation clinique à distance et la prise de décision diagnostique et thérapeutique. Cette activité s’inscrit dans le cadre du projet national de téléconsultation coordonné par le Centre d’expertise et de recherche en télémédecine et e-santé (CERTES), financé par *Geneva Digital Health Hub* (GDHH) et soutenu par le ministère de la Santé et du développement social à travers la Direction générale de la Santé et de l’hygiène publique. Les sessions de téléconsultation sont organisées deux fois par semaine, les mercredis et vendredis. Néanmoins, les situations jugées urgentes peuvent faire l’objet d’une évaluation immédiate sans délai *via* la plateforme. Dans ce cas précis, la suspicion clinique de mpox a été émise lors d’une téléconsultation reliant le Centre de santé communautaire (CSCom) de Kourémalé à l’hôpital de dermatologie de Bamako. L’équipe soignante locale a recueilli les données anamnestiques, réalisé une documentation photographique standardisée des lésions cutanées et sollicité un avis spécialisé en dermatologie en temps réel. Sur la base des éléments cliniques recueillis et conformément aux directives nationales de surveillance et de prise en charge du mpox, un prélèvement cutané a été effectué le lendemain de la télécon-sultation puis acheminé au laboratoire national de référence de l’Institut national de santé publique (INSP). L’échantillon a été soumis à une analyse par PCR en temps réel pour la confirmation diagnostique, suivie d’un séquençage viral visant à caractériser la souche en cause.

## Observation

Il s’agissait d’un homme marié de 35 ans, migrant burkinabè, travaillant depuis plus d’un an dans l’orpaillage artisanal à Kourémalé, une localité transfrontalière située à cheval entre le Mali et la Guinée, dans le cercle de Kangaba, région de Koulikoro. Durant cette période, il était séparé géographiquement de sa femme, demeurée au Burkina Faso. Il ne rapportait aucun antécédent médico-chirurgical particulier ni notion récente de déplacement hors de sa zone de résidence. L’enquête clinique a permis de documenter son origine géographique, son activité professionnelle et son contexte de résidence. En revanche, les informations relatives à d’éventuelles expositions sexuelles à risque n’ont pas pu être recueillies ou documentées de manière fiable lors de l’interrogatoire. Dans un souci de confidentialité, certaines informations sociodémographiques ont été volontairement limitées.

Le patient a consulté le 14 novembre 2025 dans un centre de santé périphérique pour une éruption cutanée fébrile évoluant depuis environ trois semaines. L’histoire clinique avait débuté par une fièvre associée à des céphalées et une asthénie, suivies 3 à 4 jours plus tard de l’apparition progressive de lésions cutanées qui se sont rapidement généralisées. Le patient ne rapportait ni prurit ni douleur associée à ces lésions.

À l’examen clinique, l’état général était conservé. Les paramètres vitaux étaient normaux. Aucune adénopathie périphérique n’était retrouvée et aucun signe d’atteinte systémique n’était noté. Les muqueuses buccales et conjonctivales étaient indemnes.

Dans le cadre du projet de téléconsultation utilisant la plateforme HUG@home, des photographies cliniques ont été transmises pour téléexpertise dermatologique. L’analyse des images mettait en évidence une éruption polymorphe diffuse touchant principalement le visage, le tronc, les membres supérieurs et les parties génitales. Les lésions étaient constituées de multiples papules, vésicules ombiliquées, pustules ainsi que de quelques lésions érosivo-croûteuses, observées simultanément à différents stades évolutifs (Fig. 1 et Fig. 2). L’atteinte faciale était particulièrement marquée, avec de nombreuses lésions disséminées sur le front, les joues, les régions périorbitaires et le menton. L’aspect clinique était fortement évocateur d’une infection par le virus mpox.

**Figure 1:**
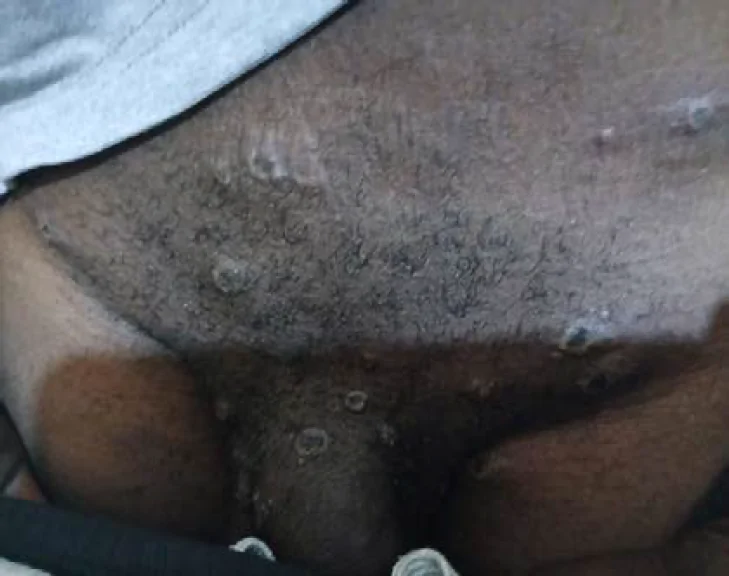
Atteinte des parties génitales

**Figure 2:**
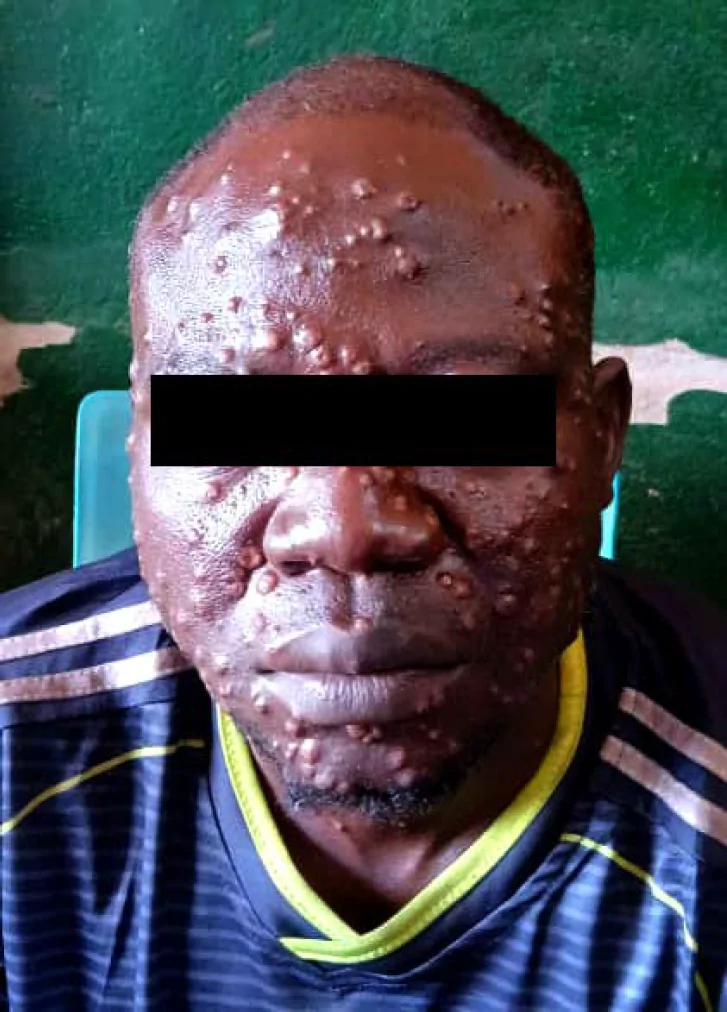
Aspect clinique initial lors de la téléconsultation montrant de multiples lésions papulo-vésiculeuses, pustuleuses et ombiliquées du visage à différents stades évolutifs

À l’issue de la téléconsultation, une suspicion de mpox a été formulée. Le patient a été immédiatement placé en isolement et a bénéficié d’un traitement symptomatique associant du paracétamol à la dose de 1 g toutes les 6 heures et des soins antiseptiques locaux visant à prévenir la surinfection des lésions cutanées. Un traitement antiviral par acyclovir avait été instauré préalablement à la confirmation du diagnostic, compte tenu des diagnostics différentiels envisagés, notamment d’autres infections virales à expression vésiculo-pustuleuse. Ce traitement a été poursuivi pendant 72 heures puis interrompu après confirmation diagnostique. Le centre de référence a été immédiatement informé afin d’organiser les investigations biologiques et les mesures de santé publique appropriées.

Un écouvillonnage des lésions cutanées a été réalisé dès le lendemain de la téléconsultation. La confirmation diagnostique a été obtenue par PCR en temps réel réalisée au laboratoire national de référence. Le délai entre la téléconsultation et l’obtention du résultat était de 72 heures, principalement en raison de l’éloignement géographique entre le centre de santé périphérique et le laboratoire de référence. L’analyse de séquençage a permis d’identifier un virus appartenant au clade IIb du virus mpox.

Après confirmation du diagnostic, le patient a été transféré, sur décision des autorités sanitaires, dans le centre d’isolement dédié à la prise en charge des maladies à potentiel épidémique du CHU du Point G, mis en place depuis la pandémie de Covid-19 et placé sous la responsabilité du service des maladies infectieuses. Un isolement strict y a été maintenu pendant quatre semaines. Les soins étaient assurés exclusivement par du personnel de santé formé, utilisant les équipements de protection individuelle recommandés. Les repas étaient fournis par le personnel soignant, tandis que les visites extérieures étaient interdites. Le centre disposait d’un accès permanent à l’eau et à l’électricité, garantissant les conditions d’hygiène et de prise en charge du patient. Les urines, les matières fécales et les autres déchets biologiques étaient éliminés selon les procédures hospitalières de gestion et de décontamination des déchets infectieux. L’évolution clinique a été favorable, avec une régression progressive des lésions suivie d’une cicatrisation complète, aboutissant à une guérison sans séquelle clinique après quatre semaines de suivi.

Des investigations épidémiologiques ont été menées afin d’identifier et de suivre les contacts ainsi que de renforcer la surveillance dans cette zone à forte mobilité transfrontalière. Au total, 48contacts ont été identifiés et suivis pendant 21 jours conformément aux recommandations en vigueur. Aucun contact n’a développé de signes évocateurs de mpox au cours de cette période de suivi.

Des activités de sensibilisation communautaire ont également été réalisées auprès des populations locales et des acteurs du système de santé. Dans les semaines suivant la clôture du suivi des contacts, trois autres cas confirmés de mpox ont toutefois été détectés dans la localité de Kourémalé. Parmi eux figurait un agent de santé ayant participé à la prise en charge du cas index. Les deux autres cas n’étaient pas des contacts directs initialement identifiés mais exerçaient leurs activités dans la même zone d’orpaillage et présentaient un contexte épidémiologique compatible avec une exposition dans ce foyer géographique.

## Discussion

Le diagnostic de mpox a été suspecté lors de la téléconsultation devant l›association d›un syndrome fébrile et d›une éruption cutanée polymorphe caractérisée par des papules, des vésicules ombiliquées, des pustules et des lésions croûteuses prédominant au visage et aux parties génitales. La confirmation diagnostique a été obtenue par PCR en temps réel sur écouvillonnage cutané, puis confirmée par séquençage identifiant un virus appartenant au clade IIb du virus mpox. Ce clade est responsable de l’épidémie multi-continentale décrite depuis 2022 et se caractérise généralement par une létalité plus faible que celle observée avec le clade I historiquement décrit en Afrique centrale [14].

Plusieurs diagnostics différentiels pouvaient être évoqués devant ce tableau clinique. La varicelle représentait le principal diagnostic alternatif en raison de l’association d’une éruption généralisée et de lésions observées à différents stades évolutifs. Cependant, certains éléments orientaient davantage vers le mpox, notamment le caractère ombiliqué des lésions, leur prédominance faciale, l’absence de prurit ainsi que l’évolution prolongée sur plusieurs semaines. Ces caractéristiques ont été rapportées comme des éléments discriminants dans plusieurs séries cliniques récentes [12,16]. D’autres diagnostics différentiels tels qu’une infection herpétique disséminée, un impétigo extensif, un molluscum contagiosum inflammatoire ou certaines formes de syphilis secondaire ont également été considérés. Toutefois, la présentation clinique globale et le contexte épidémiologique rendaient ces hypothèses moins probables [5]. Les manifestations observées chez notre patient sont globalement conformes aux données de la littérature. Classiquement, après une incubation comprise entre 5 et 21 jours, la maladie débute par un syndrome prodromique associant fièvre, céphalées, myalgies et asthénie, suivi de l’apparition de lésions cutanées évoluant progressivement vers la formation des croûtes [14,15]. Dans notre observation, la fièvre, les céphalées, l’asthénie et les lésions cutanées polymorphes correspondaient à cette présentation classique. En revanche, aucune adénopathie n’a été observée alors que ce signe est fréquemment décrit comme un élément distinctif du mpox par rapport à la varicelle [12,15]. Cette absence illustre la variabilité clinique désormais reconnue des infections à mpox, particulièrement celles liées au clade IIb [18].

L’environnement épidémiologique du patient mérite une attention particulière. Celuici exerçait une activité d’orpaillage artisanal dans une zone frontalière caractérisée par une forte mobilité des populations entre le Mali, la Guinée et plusieurs autres pays de la sousrégion. Les sites d’orpaillage constituent des espaces favorisant les interactions humaines rapprochées et la diffusion des maladies transmissibles. Bien que la source exacte de contamination n’ait pu être identifiée, plusieurs mécanismes peuvent être envisagés, notamment une transmission interhumaine à partir d’un cas non diagnostiqué, une introduction du virus par des travailleurs migrants ou une circulation communautaire silencieuse dans la zone. L’absence de voyage récent hors de la localité de résidence plaide en faveur d’une transmission locale ou régionale. Cette hypothèse est cohérente avec les observations rapportées dans plusieurs pays africains où les mouvements transfrontaliers ont été impliqués dans la diffusion du virus [13]. La détection ultérieure de trois cas confirmés supplémentaires dans la même localité apporte des arguments en faveur d’une circulation locale du virus au moment de l’identification du cas index. Bien qu’aucune chaîne de transmission formelle n’ait pu être reconstituée entre l’ensemble des cas, la survenue de ces infections dans un intervalle temporel rapproché et au sein du même environnement d’orpaillage suggère que le virus circulait probablement de manière non détectée dans cette zone. La survenue d’un cas chez un agent de santé souligne également l’importance du respect rigoureux des mesures de prévention et de contrôle des infections lors de la prise en charge des patients suspects. Dans ce contexte, la détection précoce du cas index par téléconsultation a probablement contribué au renforcement de la vigilance sanitaire locale et à l’identification rapide de nouveaux cas dans cette zone à haut risque épidémiologique.

À notre connaissance, il s’agit du premier cas confirmé de mpox rapporté au Mali et du premier cas repéré dans le cadre du programme national de téléconsultation dermatologique. Sa détection dans une zone frontalière illustre les défis posés par la surveillance des maladies émergentes dans les contextes de forte mobilité humaine. Les investigations épidémiologiques initiées après la confirmation du diagnostic, associées aux activités de sensibilisation communautaire, ont permis de renforcer les mesures de contrôle et la vigilance sanitaire dans la zone concernée.

Cette observation met également en évidence l’intérêt potentiel de la téléconsultation dermatologique dans la détection précoce des maladies infectieuses émergentes. Grâce à la plateforme HUG@home, un avis spécialisé a pu être obtenu rapidement malgré l’absence de dermatologue sur le site de prise en charge. Cette orientation diagnostic précoce a permis l’isolement du patient, la réalisation rapide des prélèvements et l’activation des dispositifs de surveillance. Plusieurs travaux ont montré que la télédermatologie constitue un outil efficace pour améliorer l’accès à l’expertise spécialisée dans les zones à ressources limitées et renforcer la détection des maladies à potentiel épidémique [11,19].

Certaines limites doivent néanmoins être soulignées. Premièrement, il s’agit d’une observation unique, ce qui limite la portée des conclusions. Deuxièmement, l’évaluation dermatologique initiale reposait sur des photographies transmises à distance dont la qualité peut influencer l’interprétation clinique. Troisièmement, malgré la documentation d’une guérison complète après quatre semaines, les données de suivi demeurent limitées et ne permettent pas d’évaluer d’éventuelles conséquences à plus long terme. Enfin, l’absence d’identification de la source de contamination empêche une meilleure compréhension des chaînes de transmission dans ce contexte. Malgré ces limites, cette observation suggère que l’intégration de la téléconsultation dermatologique dans les stratégies nationales de surveillance pourrait contribuer à améliorer la détection précoce des maladies émergentes à expression cutanée au Mali. Le renforcement des capacités des agents de santé périphériques, l’amélioration de la connectivité des structures sanitaires et l’intégration de la téléconsultation dans les dispositifs nationaux d’alerte précoce pourraient constituer des axes prioritaires pour renforcer la préparation et la réponse aux futures menaces sanitaires.

## Conclusion

Ce premier cas de mpox confirmé au Mali, détecté grâce à la télédermatologie dans une zone frontalière d’orpaillage, a conduit à la mise en œuvre rapide d’investigations épidémiologiques et à l’identification ultérieure de trois autres cas confirmés dans la même localité, suggérant une circulation locale du virus. Cette observation met en évidence le potentiel de la téléexpertise dermatologique pour la détection précoce des maladies émergentes à expression cutanée et pour le renforcement des capacités de surveillance dans les zones à accès limité aux spécialistes. Des études complémentaires seront nécessaires pour évaluer son impact sur les performances diagnostiques, la prise en charge des patients et les systèmes de surveillance à plus grande échelle.

## Consentement du patient

Le consentement verbal éclairé du patient a été obtenu pour la publication de cette observation clinique et des photographies associées. Toutes les données ont été anonymisées afin de préserver la confidentialité du patient.

## Remerciements

Les auteurs expriment leur sincère gratitude :

- à tout le personnel du Centre de santé communautaire de Kourémalé et du Centre de santé de référence de Kangaba pour leur disponibilité, leur engagement et leur excellente collaboration tout au long de la prise en charge du cas;
- à l’hôpital de dermatologie de Bamako pour son expertise dermatologique;
- au CERTES pour la coordination des activités de téléconsultation;
- au Geneva Digital Health Hub pour son soutien financier au programme de télédermatologie;
- à l’INSP pour la confirmation biologique du diagnostic;
- au Service des maladies infectieuses et tropicales du CHU du Point G pour la prise en charge spécialisée du patient dans le cadre de son isolement;
- à la Direction générale de la santé et de l’hygiène publique pour son accompagnement et son appui dans la gestion de cette investigation.

## Source de financement de la téléconsultation

Geneva Digital Health Hub (gdhub).

## Contribution des auteurs et autrices

Dramane TRAORÉ : conceptualisation, investigation, revue de la littérature, rédaction et révision du manuscrit, soumission, coordination et réalisation des téléconsultations

Bekaye TRAORÉ : suspicion du cas en tant que dermatologue expert, première correction du document

Allaye Ebe ARAMA: investigation, sollicitation de la téléconsultation, collecte des données cliniques et iconographiques, prise en charge initiale Moussa SOUGANÉ : superviseur équipes de soins, gestionnaire du système de référence et d’évacuation des patients des structures de premier niveau (CSCom) vers l’hôpital

Mahmoud CISSÉ: revue de la littérature, correction de l’article

Hamidou Souleymane TAPILY : analyse et interprétation des données cliniques, rédaction du manuscrit et validation finale du contenu scientifique

Saïdou TOURÉ : expertise dermatologique lors des téléconsultations, analyse clinique

Daouda MOLBA : investigation, réalisation du premier prélèvement biologique du patient et confirmation biologique du cas

Mouhamoudou Habib TRAORÉ : assistant à la coordination

Mahamadou Abdou DIALLO: appui à la confirmation biologique du cas

Mirana Michelle RANDRIAMBELONORO : revue et gestion du projet de télémédecine Aziza MERZOUKI: suivi et gestion du projet de télémédecine

Ibréhima GUINDO: coordination et supervision des activités du laboratoire de référence, appui technique et scientifique au processus de confirmation biologique du cas

Cheick Oumar BAGAYOKO : supervision du programme de téléconsultation, conception et révision du manuscrit

Ousmane FAYE : révision et rédaction du cas

## Déclaration de liens d’intérêt

Aucun lien d’intérêt n’a été déclaré.
