# Supplementary material for: First case of mpox detected in Mali in a gold-mining area in 2025 using teledermatology
Source: Med Trop Sante Int. 2026 Sep 11;6(3):mtsi.v6i3.2026.786. [Article in French] doi: 10.48327/mtsi.v6i3.2026.786 (PMC13697900; doi:10.48327/mtsi.v6i3.2026.786)
Supplement: Related texts

# Research article: First case of mpox detected in Mali in a gold-mining area in 2025 using teledermatology

## Introduction

Mpox (formerly monkeypox) is a zoonotic viral disease caused by an *Orthopoxvirus*. It shares clinical similarities with human smallpox but is generally less severe [8]. The infection primarily manifests clinically as skin and mucosal lesions. Transmission to humans can occur through direct contact with an infected animal or person [7]. The first human case was reported in 1970 by Ladnyi *et al.* in a nine-month-old unvaccinated boy in Basankusu, a province in the Democratic Republic of the Congo (DRC), during smallpox eradication efforts [10]. The first outbreak was reported in the DRC in 1997, when 88 cases of febrile pustular rash were identified and the virus was isolated from seven patients [9].

Mpox has long been endemic in forested regions of West and Central Africa [4]. Since 2022, the disease has spread rapidly around the world, with approximately 81,225 confirmed cases in over 100 countries [1]. While no official reports of mpox cases have been recorded in Mali, neighboring countries such as Côte d’Ivoire and Guinea-Conakry [17] have reported cases, highlighting the potential risk of the virus entering the country. Against this backdrop of regional virus circulation, the early detection of suspected cases and access to confirmatory diagnoses, particularly in rural areas with limited resources, remain major challenges for the health system. Since 2015, teledermatology has been used in Mali to improve the management of skin conditions in rural health centers [6]. It reduces disparities in access to dermatological care between urban and rural areas by facilitating remote access to specialized expertise. Using digital technologies in public health not only serves as a tool for medical care, but also as a strategic lever to strengthen the epidemiological surveillance of certain diseases in remote and hard-to-reach areas [2,3]. This is the first reported case of monkeypox in Mali that was detected via teleconsultation.

## Methods

Dermatological teleconsultations are conducted in real time via the secure HUG@home platform. The platform facilitates real-time audiovisual communication between patients receiving care at peripheral health facilities from healthcare workers and expert dermatologists. This enables remote clinical evaluation, diagnosis, and treatment decisions. This initiative is part of the national teleconsultation project coordinated by the Center for Expertise and Research in Telemedicine and e-Health (CERTES). It is funded by the Geneva Digital Health Hub (GDHH) and supported by the Ministry of Health and Social Development through the General Directorate of Health and Public Hygiene. Teleconsultation sessions are held twice a week, on Wednesdays and Fridays. However, urgent situations can be evaluated immediately via the platform. In this case, clinical suspicion of mpox arose during a teleconsultation between the Kourémalé Community Health Center (CSCom) and the Bamako Dermatology Hospital. The local healthcare team collected the patient’s medical history, took standardized photographs of the skin lesions, and requested a dermatology consultation in real time. Based on the clinical findings and in accordance with national mpox surveillance and management guidelines, the team collected a skin swab the day after the teleconsultation and sent it to the National Institute of Public Health (INSP)’s national reference laboratory. The sample underwent real-time PCR analysis for diagnostic confirmation, followed by viral sequencing to characterize the strain involved.

## Case report

The patient was a 35-year-old married man from Burkina Faso who had been working in artisanal gold mining in Kourémalé, a border town in the Kangaba Circle of the Koulikoro Region of Mali, for over a year. During this time, he was separated from his wife, who remained in Burkina Faso. He reported no significant medical or surgical history, nor any recent travel outside of his local area. The clinical investigation documented his geographic origin, occupational activity, and living situation. However, information regarding potential high-risk sexual exposures could not be reliably obtained or documented during the interview. For confidentiality reasons, certain sociodemographic details were intentionally limited. The patient presented on November 14, 2025, at a rural health center with a fever and rash that had been present for approximately three weeks. His clinical history began with a fever accompanied by headaches and asthenia. Three to four days later, he developed skin lesions that rapidly spread throughout his body. The patient reported no pruritus or pain associated with the lesions. On physical examination, the patient’s general condition was stable. Vital signs were normal. No peripheral lymphadenopathy was found, and no signs of systemic involvement were noted. The oral and conjunctival mucosa were unaffected. As part of the teleconsultation project using the HUG@home platform, clinical photographs were sent for dermatological tele-expertise. Analysis of the images revealed a diffuse, polymorphic rash that primarily affected the face, trunk, upper extremities, and genital area. The lesions consisted of multiple papules, umbilicated vesicles, pustules, and a few erosive-crusted lesions observed at various stages of development simultaneously (Fig. 1 et Fig. 2). Facial involvement was particularly pronounced, with numerous lesions scattered across the forehead, cheeks, periorbital regions, and chin. The clinical presentation was highly suggestive of mpox virus infection.

**Figure 1:** Impact on the genital area

**Figure 2:** Initial clinical presentation during the teleconsultation showing multiple papulovesicular, pustular, and umbilicated lesions on the face at various stages of development

Following the teleconsultation, mpox was suspected. The patient was immediately placed in isolation and received symptomatic treatment, including acetaminophen at a dose of 1 g every six hours and local antiseptic care to prevent secondary skin lesion infection. Given the differential diagnoses considered, particularly other viral infections presenting with vesicular-pustular lesions, anti-viral treatment with acyclovir had been initiated prior to confirmation of the diagnosis. The patient received this treatment for 72 hours and then it was discontinued following diagnostic confirmation. The reference center was immediately notified to organize the appropriate laboratory investigations and public health measures.

A swab sample of the skin lesions was collected the day after the teleconsultation. The diagnosis was confirmed by real-time PCR performed at the national reference laboratory. The time between the teleconsultation and receipt of the results was 72 hours, primarily due to the geographical distance between the peripheral health center and the reference laboratory. Sequencing analysis identified the virus as belonging to clade IIb of the mpox virus.

After the diagnosis was confirmed, the patient was transferred to the isolation center dedicated to the management of diseases with epidemic potential at Point G University Hospital. This center was established during the COVD-19 pandemic and is managed by the infectious diseases department. The patient was kept in strict isolation for four weeks. Care was provided exclusively by trained healthcare personnel wearing the recommended personal protective equipment. The nursing staff provided meals, and outside visitors were prohibited. The center had constant access to water and electricity, ensuring proper hygiene and patient care. Urine, feces, and other biological waste were disposed of in accordance with hospital procedures for managing and decontaminating waste. The patient had a favorable clinical course, with gradual lesion regression followed by complete healing. The patient made a full recovery with no clinical sequelae after four weeks of follow-up.

Epidemiological investigations were conducted to identify and monitor contacts, and to strengthen surveillance in an area with high cross-border mobility. A total of 48 contacts were identified and monitored for 21 days, in accordance with current recommendations. None of the contacts developed symptoms suggestive of mpox during this monitoring period.

Community outreach activities were also carried out among local residents and healthcare providers. However, in the weeks following the completion of contact tracing, three additional confirmed cases of mpox were detected in the town of Kourémalé. Among them was a healthcare worker who had been involved in caring for the initial case. The other two cases were not among the initial direct contacts but worked in the same gold-mining area and had an epidemiological history consistent with exposure in that geographic cluster.

## Discussion

mpox was suspected during a telemedicine consultation based on a combination of symptoms, including a fever and a rash with papules, umbilicated vesicles, pustules, and crusted lesions, which were predominantly on the face and genitals. The diagnosis was confirmed by real-time PCR on a skin swab and subsequently validated by sequencing, which identified a virus belonging to mpox clade IIb. This clade is responsible for the multiconcontinental outbreak reported since 2022 and is generally characterized by a lower case fatality rate than clade I, which has been described in Central Africa [14].

Given this clinical presentation, several differential diagnoses were possible. Chickenpox was the primary alternative diagnosis due to the presence of a generalized rash and lesions at various stages of development. However, certain features pointed more strongly toward mpox: the umbilicated nature of the lesions; their predominance on the face; the absence of pruritus; and the prolonged course, which lasted several weeks. These characteristics have been reported as distinguishing features in several recent clinical series [12,16]. Other differential diagnoses were also considered, including disseminated herpes infection, extensive impetigo, inflammatory molluscum contagiosum, and certain forms of secondary syphilis. However, the overall clinical presentation and epidemiological context rendered these hypotheses less likely [5].

The manifestations observed in our patient are generally consistent with those reported in the literature. Typically, after an incubation period of five to 21 days, the disease begins with a prodromal syndrome characterized by fever, headache, myalgia, and asthenia. This is followed by the appearance of skin lesions that gradually progress to crust formation [14,15]. In our case, the fever, headache, asthenia, and polymorphic skin lesions were consistent with this classic presentation. However, no lymphadenopathy was observed, despite it being frequently described as a distinguishing feature of mpox compared to chickenpox [12,15]. This absence illustrates the clinical variability now recognized in mpox infections, particularly those associated with clade IIb [18].

The patient’s epidemiological context warrants special attention. He was involved in artisanal gold mining in a border region with high population mobility between Mali, Guinea, and other countries in the area. Gold mining sites facilitate close human contact and the spread of communicable diseases. While the exact source of the patient’s infection could not be identified, several possible mechanisms can be considered: human-to-human transmission from an undiagnosed case; introduction of the virus by migrant workers; or silent community circulation in the area. The absence of recent travel outside the patient’s place of residence supports the hypothesis of local or regional transmission. This hypothesis aligns with observations reported in several African countries, where cross-border movement has been associated with the spread of the virus [13]. The subsequent detection of three additional confirmed cases in the same locality provides evidence of local virus circulation at the time the index case was identified. While no formal chain of transmission could be reconstructed among all the cases, the occurrence of these infections within a short timeframe and within the same gold-mining community suggests that the virus was likely circulating undetected in the area. The occurrence of a case among a healthcare worker underscores the importance of strictly adhering to infection prevention and control measures when caring for suspected patients. In this context, the early detection of the index case via telemedicine likely contributed to heightened local health surveillance and the rapid identification of new cases in this epidemiologically high-risk area. To our knowledge, this is the first confirmed case of monkeypox reported in Mali, as well as the first case identified through the national dermatological teleconsultation program. The detection of the virus in a border area highlights the challenges of monitoring emerging diseases in areas with high human mobility. Epidemiological investigations, combined with community awareness activities, were initiated after the diagnosis was confirmed and have helped strengthen control measures and public health surveillance in the affected area. This case highlights the potential value of dermatological teleconsultations for the early detection of emerging infectious diseases. Thanks to the HUG@home platform, a specialist opinion was obtained quickly despite the absence of a dermatologist at the treatment site. This early diagnostic guidance enabled the patient’s isolation, the prompt collection of specimens, and the activation of surveillance measures. Several studies have demonstrated the effectiveness of teledermatology in improving access to specialized expertise in areas with limited resources and enhancing the detection of diseases with epidemic potential [11,19].

However, certain limitations must be noted. First, this is a single-case report, which limits the scope of the conclusions. Second, the initial dermatological evaluation relied on photographs transmitted remotely, whose quality may have influenced the clinical interpretation. Third, although the patient made a full recovery after four weeks, follow-up data is limited and does not allow for an assessment of potential long-term consequences. Finally, the failure to identify the source of contamination hinders our understanding of transmission chains in this context.

Despite these limitations, this case suggests that integrating dermatological teleconsultation into national surveillance strategies could help improve the early detection of emerging diseases with cutaneous manifestations in Mali. Strengthening the capacity of frontline health workers, improving connectivity among health facilities, and integrating telemedicine into national early-warning systems could be priority areas for enhancing preparedness and response to future health threats.

## Conclusion

This first confirmed case of mpox in Mali, detected through teledermatology in a border area where gold mining occurs, led to the rapid implementation of epidemiological investigations and the subsequent identification of three other confirmed cases in the same locality, suggesting local circulation of the virus. This finding highlights the potential of dermatological tele-expertise for the early detection of emerging diseases with cutaneous manifestations and for strengthening surveillance capabilities in areas with limited access to specialists. Further studies will be needed to assess its impact on diagnostic performance, patient management, and surveillance systems on a larger scale.

## Patient consent

Verbal informed consent was obtained from the patient for the publication of this clinical case report and the associated photographs. All data were anonymized to protect patient confidentiality.

## Acknowledgments

The authors express their sincere gratitude to:

- all staff at the Kourémalé Community Health Center and the Kangaba Referral Health Center for their availability, commitment, and excellent collaboration throughout the management of this case;
- the Bamako Dermatology Hospital for its dermatological expertise;
- the CERTES for coordinating the teleconsultation activities;
- the Geneva Digital Health Hub for its financial support of the teledermatology program;
- the INSP for the laboratory confirmation of the diagnosis;
- the Department of Infectious and Tropical Diseases at the Point G University Hospital for the specialized care of the patient during his isolation;
- the General Directorate of Health and Public Hygiene for its guidance and support in managing this investigation.

## Source of funding for the teleconsultation

Geneva Digital Health Hub (gdhub).

## Authors’ contributions

Dramane TRAORÉ: conceptualization, investigation, literature review, drafting and revising the manuscript, submission, coordination and conduct of teleconsultations

Bekaye TRAORÉ: identification of the suspected case as an expert dermatologist, initial editing of the document

Allaye Ebe ARAMA: investigation, requesting the teleconsultation, collection of clinical and imaging data, initial management

Moussa SOUGANÉ: supervisor of care teams; manager of the patient referral and transfer system from primary-care facilities (CSCom) to the hospital

Mahmoud CISSÉ: literature review; editing of the article

Hamidou Souleymane TAPILY: analysis and interpretation of clinical data, drafting of the manuscript, and final validation of the scientific content Saïdou TOURÉ: dermatological expertise during teleconsultations, clinical analysis

Daouda MOLBA: investigation, collection of the patient’s first biological sample, and biological confirmation of the case

Mouhamoudou Habib TRAORÉ: coordination assistant

Mahamadou Abdou DIALLO: support for the biological confirmation of the case

Mirana Michelle RANDRIAMBELONORO: review and management of the telemedicine project Aziza MERZOUKI: monitoring and management of the telemedicine project

Ibréhima GUINDO: coordination and supervision of reference laboratory activities; technical and scientific support for the biological case confirmation process

Cheick Oumar BAGAYOKO: supervision of the teleconsultation program; manuscript design and revision

Ousmane FAYE: revision and drafting of the case report

## Conflicts of interest

The authors declare no conflicts of interest.
